# Phosphorus requirements of laying Japanese quails

**DOI:** 10.1016/j.psj.2025.105346

**Published:** 2025-05-28

**Authors:** Mehran Mehri, Mohsen Sargazi, Mahmoud Ghazaghi, Farzad Bagherzadeh-Kasmani, Morteza Asghari-Moghadam, Hamid-Reza Behboodi, Mohammad Rokouei

**Affiliations:** a Department of Animal Sciences, Faculty of Agriculture, University of Zabol, Sistan, 98661-5538, Iran; b Behbood Poultry Nikaan Shomal Company, Tehran, Iran

**Keywords:** Alkaline phosphatase, eggshell, laying quail, performance, requirements

## Abstract

Available phosphorus (AP) is a crucial nutrient for laying quails, influencing eggshell quality and overall production performance. This study investigated the optimal levels of dietary AP in laying quails from 10 to 16 weeks of age. A total of 375 laying quails were used in a completely randomized design with five experimental diets (0.25, 0.30, 0.35, 0.40, and 0.45 % AP) and five replicates. Dietary AP levels did not affect feed intake, feed conversion ratio (FCR), egg mass (EM), or egg production (EP) percentage (*P* > 0.05). Egg characteristics were influenced by AP levels, with significant effects observed for egg width (*P* = 0.011), egg volume (*P* = 0.025), and shell thickness (*P* = 0.041), all displaying linear trends. Serum alkaline phosphatase (ALP) concentration exhibited a nonlinear response, decreasing with increasing AP levels, supporting an estimated AP requirement of 0.357 % based on the broken-line model. Estimated P requirements indicated that optimal AP levels for FCR, EP, and ALP were 0.374 %, 0.383 %, and 0.357 %, respectively. Overall, an AP level of approximately 0.38 % was identified as optimal for maximizing egg production while maintaining efficient feed utilization and eggshell quality in laying quails.

## Introduction

Laying quail requires a well-balanced diet to maintain optimal egg production and quality. Among the essential nutrients, available phosphorus (AP) plays a crucial role in various physiological processes, including eggshell formation and bone mineralization. The dietary requirements for AP in laying quails have been a subject of ongoing research, as optimal levels can significantly impact production performance and egg quality (Aguda et al., 2015; Rossetto, et al., 2019).

Phosphorus is a crucial nutrient for poultry, including quail, as it plays a vital role in various physiological functions, including bone development and energy metabolism. However, a significant portion of phosphorus in plant-based feeds, such as cereal grains and oilseed meals, is present as phytic acid, which has low bioavailability for birds due to their limited endogenous phytase activity (Pallauf and Rimbach, 1997). Phytic acid can chelate essential minerals, further reducing their availability, which can lead to deficiencies if not managed properly in diets (Singh and Satyanarayana, 2015). Therefore, it is essential to consider the levels of AP in quail diets to ensure adequate phosphorus utilization and prevent deficiencies. Supplementation with microbial phytase can enhance phosphorus bioavailability by hydrolyzing phytic acid, thus improving nutrient absorption and reducing environmental phosphorus pollution (Cangussu, et al., 2018).

Previous studies have investigated the effects of varying AP levels on laying quail performance. Research has shown that AP requirements may differ depending on the age and production stage of the birds. For instance, calcium and AP requirements for egg production can vary between the beginning and end of the egg-production cycle (Ribeiro, et al., 2016). Recent studies have explored the effects of different AP levels on various production parameters in laying quails. Ribeiro, et al. (2016) reported that dietary AP levels influenced egg quality parameters and suggested that higher levels might be beneficial for maintaining eggshell quality in older laying quails.

The interaction between calcium (Ca) and AP is also a critical factor in quail nutrition. These minerals work together during absorption, metabolism, and excretion processes. Maintaining an appropriate balance between Ca and AP levels in quail diets is essential for optimal nutrient utilization and egg quality (Nascimento, et al., 2019). Research indicates that varying levels of Ca and AP can significantly influence egg weight, shell quality, and overall production performance in quails. For instance, higher dietary Ca levels have been associated with improved eggshell percentage and strength, while adequate AP levels are necessary for efficient feed conversion and bone health (Amoah, et al., 2012; Garcia, et al., 2000; Vieira, et al., 2012).

Although previous studies have explored the effects of Ca, phytase, and P levels on egg production and bone mineralization in laying quails, many of these investigations have confounded the effects of AP with simultaneous variations in dietary Ca or inclusion of exogenous phytase. This makes it difficult to isolate the specific contribution of AP to performance and egg quality outcomes. Furthermore, there is limited data defining the optimal AP requirements specifically for laying quails between 10 and 16 weeks of age — a critical early laying phase. Therefore, this study was designed to address this gap by systematically evaluating the effects of graded levels of dietary AP under fixed calcium levels and without phytase supplementation. This approach provides a clearer understanding of AP's standalone role in early-laying quails and establishes reference values for practical diet formulation.

## Materials and Methods

### Ethics Statement

This study followed Iranian Council for Animal Care guidelines and had its protocol approved by the Research Animal Ethics Committee of the University of Zabol (AEUOZ-2012-BR).

### Chemical Analysis

Prior to the experiment, all feed ingredients used in the experimental diets were analyzed for dry matter (DM; method 930.15, AOAC, 2006), ash content (method 942.05, AOAC, 2006), crude fiber (method 978.10, AOAC, 2006), ether extract (method 2003.05, AOAC, 2006), Ca (method 934.01,AOAC (2006), and crude protein (CP; method 990.03, AOAC, 2006).

### Birds Management and Experimental Diets

In this dose-response assay, the birds received standard diet based on corn and soybean meal to meet all nutritional requirements according to NRC (1994) recommendations from hatch up to 8 weeks of age. In eight weeks of experiment, a total of 375 laying quails (255 ± 9.06 g) were fed five experimental diets (0.25, 0.30, 0.35, 0.40, and 0.45 % AP) with five replicates, and 15 birds each in a completely randomized design to 16 weeks of age (the first 2 weeks adaptation). A basal diet, deficient in AP, was formulated (Table 1) and then four additional experimental diets were developed with 0.05 % increments in AP. The laying quails had free access to feed and water during the experiment. Room temperature was kept at 20 ± 2.2 °C, and humidity at 55 ± 3.5 %. An 18L:6D photoperiod was maintained throughout the experiment.

**Table 1:** Composition of experimental diets.

Ingredient	Available phosphorus (%)				
	0.25	0.30	0.35	0.40	0.45
Corn, Grain	57.11	57.11	57.11	57.11	57.11
Soybean meal	31.43	31.43	31.43	31.43	31.43
Limestone	6.04	5.90	5.76	5.62	5.48
Soybean oil	2.70	2.70	2.70	2.70	2.70
DL-methionine	0.15	0.15	0.15	0.15	0.15
Sand	1.00	0.89	0.78	0.67	0.56
Di-calcium phosphate	0.48	0.73	0.98	1.23	1.48
NaHCO_3_	0.39	0.39	0.39	0.39	0.39
NaCl	0.20	0.20	0.20	0.20	0.20
Mineral premix^a^	0.25	0.25	0.25	0.25	0.25
Vitamin premix^b^	0.25	0.25	0.25	0.25	0.25
**Nutrient composition**					
Calculated ME (Kcal/kg)^d^	2900	2900	2900	2900	2900
CP (%)^e^	20.0	20.0	20.0	20.0	20.0
Methionine (%)	0.45	0.45	0.45	0.45	0.45
Arginine (%)	1.32	1.32	1.32	1.32	1.32
Lysine (%)	1.05	1.05	1.05	1.05	1.05
Methionine + Cysteine (%)	0.76	0.76	0.76	0.76	0.76
Threonine (%)	0.75	0.75	0.75	0.75	0.75
Tryptophan (%)	0.23	0.23	0.23	0.23	0.23
Ca (%)^e^	2.50	2.50	2.50	2.50	2.50
P available (%)	0.25	0.30	0.35	0.40	0.45
K (%)	0.81	0.81	0.81	0.81	0.81
Na (%)	0.21	0.21	0.21	0.21	0.21
Cl (%)	0.16	0.16	0.16	0.16	0.16
DEB (mEq/kg)^c^	250	250	250	250	250

a Mineral premix provided per kilogram of diet: Mn (from MnSO4·H2O), 65 mg; Zn (from ZnO), 55 mg; Fe (from FeSO4·7H2O), 50 mg; Cu (from CuSO4·5H2O), 8 mg; I [from Ca (IO3)2·H2O], 1.8 mg; Se, 0.30 mg; Co (from Co2O3), 0.20 mg; Mo, 0.16 mg.

b Vitamin premix provided per kilogram of diet: vitamin A (from vitamin A acetate), 11,500 IU; cholecalciferol, 2,100 IU; vitamin E (from dl-α-tocopheryl acetate), 22 IU; vitamin B12, 0.60 mg; riboflavin, 4.4 mg; nicotinamide, 40 mg; calcium pantothenate, 35 mg; menadione (from menadione dimethylpyrimidinol), 1.50 mg; folic acid, 0.80 mg; thiamine, 3 mg; pyridoxine, 10 mg; biotin, 1 mg; choline chloride, 560 mg; ethoxyquin, 125 mg.

c Dietary Electrolyte Balance: represents dietary Na +*K* − Cl in mEq/kg of diet.

d calculated based on standard feed composition tables, not measured through metabolism trials.

e Analyzed values.

### Productive Performance

The daily count of eggs and their individual weights (EW, g), along with the weekly feed intake (FI, g/bird), were measured throughout the trial. The average egg production (EP, %) was determined by dividing the total number of eggs produced by the number of laying quails. Egg mass (EM) was calculated as the product of egg weight and EP percentage. The feed conversion ratio (FCR) was expressed as the total FI per unit of EM (Shariat Zadeh, et al., 2020).

### Egg Characters

During the final week of the trial, six consecutive eggs per replicate cage (*n* = 30 eggs per treatment) were collected for egg quality assessment. Egg length and width were measured using a digital micrometer (Mitutoyo Corporation, Model 293-821, Japan) with an accuracy of 0.01 mm. The length was recorded as the longest axis of the egg, while the width was measured at the broadest part of the egg. Egg volume (cm³) was estimated using the formula (Angel Daniel, et al., 2022):

$$V=0.485\times L\times W^{2}/1000$$

where *L* is the longitudinal diameter (mm); *W* is the transverse diameter (mm).

Eggshell thickness (µm) was determined using a digital micrometer (accuracy: 1 µm). Measurements were taken at three locations: the broad end, the narrow end, and the equatorial region of the shell. The final shell thickness value was obtained by averaging the three measurements (Angelovič and Zelenakova, 2024).

### Serum Alkaline Phosphatase

At the end of the experimental period (week 16), three birds per replicate cage (*n* = 15 birds per treatment) were randomly selected for blood collection via brachial vein puncture. Blood samples were collected in the sterile syringes. Serum alkaline phosphatase (ALP) was measured using a Pars-Azmoun (Tehran, Iran) commercial kit after a 30-minute room temperature blood clot and a 10-minute centrifugation at 3,000 rpm.

### Statistical Analysis

Data were analyzed using the MIXED procedure in SAS (2002). A repeated measures model was used to evaluate the main effects of dietary AP level, time (weeks), and their interaction (AP × time). The experimental unit (replicate pen) was considered the subject for repeated measurements. Time was treated as a repeated factor, and various covariance structures (i.e., compound symmetry) were tested and least square means were compared using the Tukey adjustment, and significance was declared at *P* < 0.05.

Phosphorus requirements were estimated using two-slope broken line regression models, using NLIN procedure in SAS, as outlined by Khosravi, et al. (2016):

Linear ascending (or descending)-linear descending (or ascending):

Y=L+U×(R−−X)×(X<R)+V×(X−−R)×(X>R)

where Y represents the bird's response; L denotes the asymptote for the first segment; U and V correspond to the slopes of the first and second segments, respectively, indicating an increasing or decreasing trend; and R represents the breakpoint, which is considered the P requirement.

## RESULTS

### Performance Parameters

The effects of dietary available phosphorus (AP) levels on the performance of laying quails are presented in Table 2. There were no significant differences (*P* > 0.05) in feed intake, feed conversion ratio, egg mass, and egg production percentage among the dietary treatments. The feed intake ranged from 35.8 g/bird to 37.9 g/bird, while the feed conversion ratio varied between 3.71 and 4.22. Egg mass showed a numerical increase up to 10.4 g at 0.40 % AP but was not statistically significant (*P* = 0.409). Egg weight was also unaffected by AP levels (*P* = 0.488), but a significant effect of time was observed (*P* < 0.001).

**Table 2:** Laying quail performance over time in response to different dietary levels of available phosphorus (AP; %).

Response		AP (%)				SEM	Probabilities				
	0.25	0.30	0.35	0.40	0.45		Model	Time	*T* × Time	L	Q
Feed intake (g/b)	36.8	36.4	36.9	37.9	35.8	0.71	0.339	0.654	0.659	0.562	0.931
Feed conversion ratio	3.87	3.79	3.71	3.86	4.22	0.22	0.535	0.628	0.455	0.753	0.187
Egg mass (g)	9.77	9.93	10.1	10.4	8.94	0.53	0.409	0.754	0.690	0.829	0.225
Egg weight (g)	12.8	12.8	12.6	13.0	12.7	0.18	0.488	< 0.001	0.379	0.560	0.353
Egg production (%)	56.1	56.7	58.3	57.9	55.0	1.59	0.608	0.119	0.239	0.936	0.173

The mean value was determined based on 15 birds per replicate (total of 75 birds per treatment).

L: linear effect; Q: quadratic effect.

### Egg Characteristics

The impact of dietary AP on egg characteristics is summarized in Table 3. Egg length was not significantly affected by AP levels (*P* = 0.270); however, a significant linear effect was detected (*P* = 0.007). Egg width exhibited a significant treatment effect (*P* = 0.011), with a linear trend (*P* = 0.029). Similarly, egg volume showed significant treatment (*P* = 0.025) and linear effects (*P* = 0.001), indicating a decrease in volume with increasing AP levels. Shell thickness was significantly influenced by dietary AP (*P* = 0.041), with a strong linear relationship (*P* < 0.001).

**Table 3:** Egg characteristics of laying quails over time in response to different dietary levels of available phosphorus (AP; %).

Response		AP (%)				SEM	Probabilities				
	0.25	0.30	0.35	0.40	0.45		Model	Time	*T* × Time	L	Q
Length (mm)	33.9	33.7	33.4	33.6	33.8	0.17	0.270	0.007	0.183	0.644	0.050
Width (mm)	26.2	26.2	25.9	25.9	26.3	0.08	0.011	0.029	0.741	0.866	0.007
Volume (cm^3^)	11.3	11.3	10.9	11.0	11.4	0.12	0.035	< 0.001	0.369	0.686	0.296
Shell thickness (µm)	0.231	0.232	0.229	0.231	0.235	0.001	0.041	< 0.001	0.617	0.406	0.992

The mean value was determined based on 30 eggs per treatment).

L: linear effect; Q: quadratic effect.

### Serum Alkaline Phosphatase Concentration

**Fig. 1:**
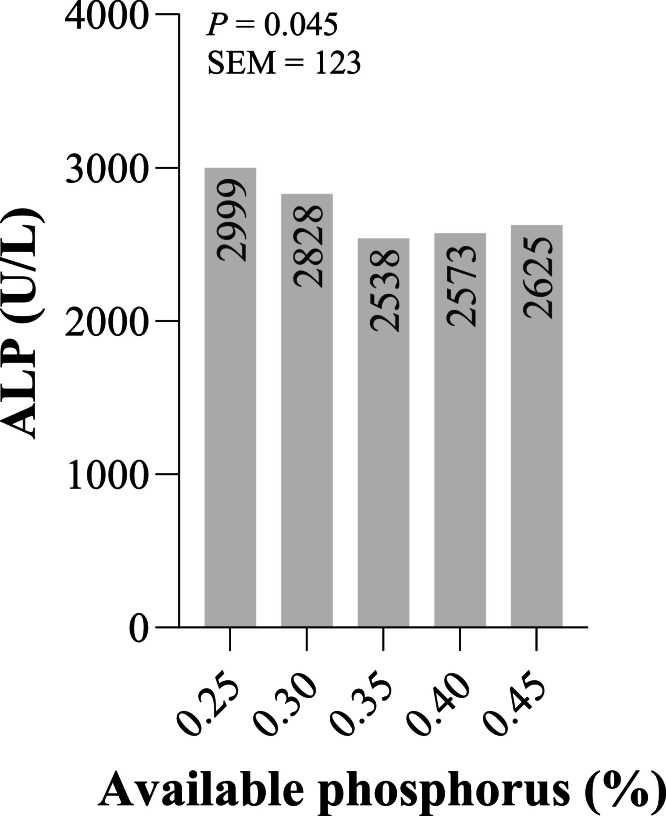
Serum concentration of alkaline phosphatase (ALP) of laying quails (*n* = 15 birds per treatment) at different dietary levels of available phosphorus (AP; %).

The serum concentration of alkaline phosphatase (ALP) at different AP levels is illustrated in Figure 1. The results indicate a nonlinear response, with ALP activity decreasing as dietary AP levels increased, supporting the estimated AP requirement of 0.357 % based on the broken-line model.

### Estimated Phosphorus Requirements

Table 4 presents the estimated AP requirements of laying quails using two-slope broken-line models. The estimated requirement for FCR was determined to be 0.374 % AP (R² = 0.999). For EP, the optimal AP level was estimated at 0.383 % (R² = 0.982), while for optimal ALP, the estimated requirement was 0.357 % AP (R² = 0.985).

**Table 4:** Estimated available phosphorus requirements (R) of laying quails using two-slope broken line models.

Response	Model	R	Standard error (±)	95 % Confidence Interval	R^2^
FCR^1^	*y* = 3.67 + 1.58 × (0.374 - x) + 7.232 × (x – 0.374)	0.374	0.001	0.372-0.376	0.999
EP^2^	*y* = 0.5886 – 0.22 × (0.383 - x) – 0.574 × (x – 0.383)	0.383	0.009	0.365-0.401	0.982
ALP^3^	*y* = 2528 + 4608 × (0.357 - x) – 1041 × (x – 0.357)	0.357	0.020	0.318-0.396	0.985

1 Feed conversion ratio.

2 Egg production.

3 Alkaline phosphatase.

## Discussion

While it is recognized that poultry, including laying quail, possesses Ca and P reserves within bone tissue that can be mobilized during periods of dietary insufficiency, the present study was carefully designed to minimize this potential confounding effect. The duration of the trial (from 10 to 16 weeks of age) corresponds to the early laying phase, during which dietary demands for P increase due to eggshell formation and bone remodeling. Furthermore, because the study spanned several weeks, cumulative performance parameters (egg production, feed efficiency), as well as egg characteristics, were used as indicators of long-term P adequacy rather than short-term compensation via skeletal reserves. This timeframe is sufficient to observe production and mineralization responses that reflect actual dietary P adequacy under standard feeding conditions.

This study examined the effects of varying dietary available phosphorus (AP) levels on the performance, egg characteristics, and serum alkaline phosphatase (ALP) concentration of laying quails. The objective was to determine the optimal AP requirement for balancing productivity, egg quality, and physiological responses. The results indicate a complex interaction between dietary AP and various parameters, with significant effects observed on egg characteristics and enzyme activity, but minimal impact on overall performance indicators, such as FI and EP percentage within the tested range.

### Performance Parameters

The absence of significant differences in FI, FCR, EM, and EP percentage across the dietary AP levels was somewhat unexpected but not entirely surprising. Phosphorus plays a critical role in metabolic processes, including energy utilization and protein synthesis. However, the relatively stable performance observed in laying quails across the tested AP range suggests several potential explanations. One possibility is that the lowest AP level used in the study may have already met the birds' basal metabolic needs, preventing any noticeable variations in performance. Additionally, quails may possess physiological mechanisms to compensate for fluctuations in AP availability, such as increased P absorption efficiency in lower AP groups or mobilization of P from bone reserves to sustain egg production (Ribeiro, et al., 2016; Souza, et al., 2017). The skeleton serves as a reservoir for Ca and P, crucial for eggshell formation, and physiological adaptations occur to ensure skeletal integrity and egg quality throughout the laying cycle in poultry (Garcia-Mejia, et al., 2024). Although this compensation helps maintain performance, it could have long-term implications for bone health, which were not evaluated in this study. The numerical increase in EM at 0.40 % AP, while not statistically significant, suggests a potential positive effect of higher AP levels.

### Egg Characteristics

In contrast to performance parameters, egg characteristics exhibited a more pronounced response to dietary AP levels. Significant treatment effects on egg width and egg volume, along with observed linear trends, indicate that AP levels can influence egg size and shape. Interestingly, the observed decrease in egg volume with increasing AP levels suggests a possible shift in the proportions of egg components rather than an overall reduction in egg content, warranting further investigation into egg composition. A particularly notable finding was the significant influence of dietary AP on shell thickness, with a strong linear relationship observed. The finding supports the known function of P in Ca metabolism and bone mineralization. Increased AP levels likely enhanced Ca deposition in the shell gland, resulting in thicker, stronger eggshells. Research indicates that higher dietary AP levels positively influence the expression of Ca transporters in the shell gland, facilitating better Ca absorption and deposition during eggshell formation (Costa, et al., 2007; Garcia, et al., 2000). Consequently, optimizing dietary AP not only improves eggshell strength but also contributes to the economic viability of quail farming by minimizing losses due to breakage and enhancing the marketability of the eggs (Costa, et al., 2010; de Souza, et al., 2016).

### Serum Alkaline Phosphatase Activity

Serum ALP concentration provides valuable insights into bone metabolism and phosphorus utilization in laying quails. The nonlinear response of ALP activity, with decreasing levels as dietary AP increased, supports the hypothesis that P intake regulates bone metabolism. At lower AP levels, quails may rely more on P mobilization from bone reserves, leading to increased bone turnover and elevated ALP activity. As dietary AP increases, the reliance on bone mobilization decreases, reflected in lower ALP activity (Cannalire, et al., 2023; Cheng and Zhao, 2023). This response supports the hypothesis that phosphorus intake regulates bone metabolism in quails. The mobilization of phosphorus from bone reserves is crucial for maintaining bone health and egg production, particularly when dietary phosphorus is insufficient (Costa, et al., 2007; Tang, et al., 2024). Thus, monitoring serum ALP levels can provide insights into the nutritional status and metabolic health of laying quails, which is essential for optimizing their production performance. The estimated AP requirement of 0.357 % based on the broken-line model further supports this interpretation, suggesting that this level represents the threshold at which bone mobilization is minimized, and ALP activity is optimized. However, ALP activity can also be influenced by factors such as age, disease status, and dietary components, necessitating a holistic approach when interpreting these data alongside performance and egg quality parameters.

### Estimated Phosphorus Requirements

The P requirements estimated through broken-line regression models highlight the critical role of dietary AP optimization in laying quail diets. Specifically, the estimated AP levels for FCR at 0.374 %, EP at 0.383 %, and ALP activity at 0.357 %, all cluster around 0.38 %. This convergence across distinct performance and physiological parameters strongly supports the conclusion that approximately 0.38 % AP is optimal for achieving high egg production, efficient feed utilization, and reduced bone mobilization.

Formulating diets to supply around 0.38 % AP appears to provide the best balance between performance outcomes, eggshell quality, and bone metabolism. Adequate AP levels are essential for enhancing shell strength and minimizing egg breakage—an important consideration for commercial quail production. The robustness of these estimates is further confirmed by the high R² values from the broken-line models, indicating reliable model fit and predictive capacity.

Notably, the estimated AP values in this study exceed the NRC (1994) recommendation of 0.35 %. The confidence intervals for our estimates reinforce the biological relevance of this difference and suggest that modern strains of laying quails may have higher P requirements than previously recognized. Several factors may contribute to this divergence. First, NRC (1994) guidelines are based on older quail genotypes with lower productivity, while current commercial strains have been genetically selected for improved egg production and thus possess higher nutrient demands. Second, the NRC (1994) values are often derived from factorial approaches, which may not fully account for physiological responses under dynamic, modern production systems. Third, differences in dietary composition, management practices, housing systems, and health status between historical reference conditions and today’s operations may influence P utilization and requirements.

Interestingly, a similar trend has been observed regarding Ca requirements in modern quail strains, where current estimates also exceed those provided by the NRC (1994). Recently, we showed that immune responses in laying quails require higher calcium levels (∼0.852 %) than those needed for performance and other physiological traits (Ahmadi, et al., 2025), with optimal levels for bone breaking strength ranging from 0.75 to 0.83 %, respectively (Pourmollaei, et al., 2024). This parallel underscores the broader need to re-evaluate and update mineral nutrition guidelines to align with genetic advancements and contemporary production systems.

In summary, our findings emphasize that an AP level of approximately 0.38 % is optimal for maintaining productivity, egg quality, and metabolic health in laying quails. This level should be targeted in diet formulation to minimize economic losses due to egg breakage and to support long-term skeletal health. The study further confirms that dietary AP has a significant impact on key traits such as shell thickness and ALP activity, even though overall performance metrics remained stable across the tested AP range. Future investigations will explore the long-term implications of dietary AP on bone mineralization, P digestibility, and egg composition, as well as interactions with nutrients like vitamin D and the use of exogenous enzymes. Ultimately, careful change of AP levels can contribute to improved efficiency, sustainability, and profitability in laying quail production.

## Declaration of competing interest

The authors declare that they have no known competing financial interests or personal relationships that could have appeared to influence the work reported in this paper.
